# Multiparameter MRI-based radiomics nomogram for preoperative prediction of brain invasion in atypical meningioma:a multicentre study

**DOI:** 10.1186/s12880-024-01294-5

**Published:** 2024-06-05

**Authors:** Jinna Yu, Xin Kong, Dong Xie, Fei Zheng, Chao Wang, Dan Shi, Cong He, Xiaohong Liang, Hongwei Xu, Shouwei Li, Xuzhu Chen

**Affiliations:** 1 Department of Radiology, Shaoxing Second Hospital, Shaoxing, P.R. China; 2 Department of Radiology, Beijing Tiantan Hospital, Capital Medical University, No.119 South Fourth Ring West Road, Fengtai District, Beijing, 100070 P. R. China; 3 Department of Radiology, The Second Affiliated Hospital, Zhejiang University School of Medicine, Hangzhou, P.R. China; 4 Department of Pathology, Shaoxing Second Hospital, Shaoxing, P.R. China; 5 Department of Neurosurgery, SanBo Brain Hospital, Capital Medical University, Beijing, P. R. China

**Keywords:** Brain invasion, Peritumoral edema, Atypical meningiomas, Magnetic resonance imaging, Radiomics

## Abstract

**Objective:**

To develop a nomogram based on tumor and peritumoral edema (PE) radiomics features extracted from preoperative multiparameter MRI for predicting brain invasion (BI) in atypical meningioma (AM).

**Methods:**

In this retrospective study, according to the 2021 WHO classification criteria, a total of 469 patients with pathologically confirmed AM from three medical centres were enrolled and divided into training (*n* = 273), internal validation (*n* = 117) and external validation (*n* = 79) cohorts. BI was diagnosed based on the histopathological examination. Preoperative contrast-enhanced T1-weighted MR images (T1C) and T2-weighted MR images (T2) for extracting meningioma features and T2-fluid attenuated inversion recovery (FLAIR) sequences for extracting meningioma and PE features were obtained. The multiple logistic regression was applied to develop separate multiparameter radiomics models for comparison. A nomogram was developed by combining radiomics features and clinical risk factors, and the clinical usefulness of the nomogram was verified using decision curve analysis.

**Results:**

Among the clinical factors, PE volume and PE/tumor volume ratio are the risk of BI in AM. The combined nomogram based on multiparameter MRI radiomics features of meningioma and PE and clinical indicators achieved the best performance in predicting BI in AM, with area under the curve values of 0.862 (95% CI, 0.819–0.905) in the training cohort, 0.834 (95% CI, 0.780–0.908) in the internal validation cohort and 0.867 (95% CI, 0.785–0.950) in the external validation cohort, respectively.

**Conclusions:**

The nomogram based on tumor and PE radiomics features extracted from preoperative multiparameter MRI and clinical factors can predict the risk of BI in patients with AM.

**Supplementary Information:**

The online version contains supplementary material available at 10.1186/s12880-024-01294-5.

## Introduction

Atypical meningioma (AM), one of 15 subtypes of meningioma, is classified as a WHO grade 2 tumor with a certain degree of aggressiveness, with an invasiveness between that of benign and malignant meningioma; it accounts for approximately 24.5% of all meningiomas [1], and it has a higher risk of postoperative recurrence than WHO grade 1 meningiomas [2–4]. Brain invasion (BI) refers to the presence of meningioma tissue in the adjacent brain tissue without a separate connective tissue layer and tumor cells infiltration into the brain parenchyma in irregular tongue-like projections without an intervening pia mater [2, 5]. BI was clarified in the 2016 revision of the CNS WHO 4 classification as an independent histological criterion for the diagnosis of AM [6]. The 2021 WHO CNS 5, the latest revision, emphasises BI as a pathological diagnostic criterion for AM and applies to any potential subtype [7].

BI in meningiomas has a distinct clinical significance and is independently associated with tumor progression, recurrence and poor prognosis [2, 8–11]. The presence of BI is closely related to the choice of surgical technique, such as the application of intraoperative navigation, expansion of surgical excision range, etc [12, 13]. . BI makes surgery far more difficult and may have negative implications on functional outcome. In addition, BI is a risk factor for preoperative epileptic seizure and postoperative bleeding [14–16]. Therefore, accurately identifying BI in meningioma is of important clinical significance. AM is more likely to recur and has a worse prognosis than WHO grade 1 meningioma [17]. Additionally, the prevalence of AM has increased to 20–35% following the use of BI as an independent diagnostic criterion for the condition [17, 18], necessitating studying AM as a distinct research object. Currently, the gold standard for the diagnosis of BI is the histopathological examination, but this is an invasive procedure that cannot capture the associated changes in real time. It is therefore important to develop practical means to prospectively and noninvasively determine BI in AM.

Several previous studies explored the correlation between BI and imaging features, such as peritumoral edema (PE), enhanced heterogeneity and irregular tumor shape, which were the independent risk predictors of BI [19–23]. Radiomics is an emerging image processing method developed in recent years that allows high-throughput extraction and quantitative analysis of radiomics features in images that cannot be identified by the naked eye [24, 25]. Although there are several radiomics studies on BI prediction of meningiomas [12, 26], there is a lack of radiomics studies on BI prediction of atypical meningiomas. Since the imaging features of atypical meningiomas are obviously different from those of meningiomas, it is necessary to develop a special radiomics study to establish a prediction model of BI for atypical meningiomas.

Therefore, using multicentre data, the aim of this study was to analyze the MRI-based radiomics features of AM tumors and PE, to develop a nomogram for predicting BI in AM patients, and to compare the predictive performance of different models.

## Materials and methods

### Patients

**Fig. 1:**
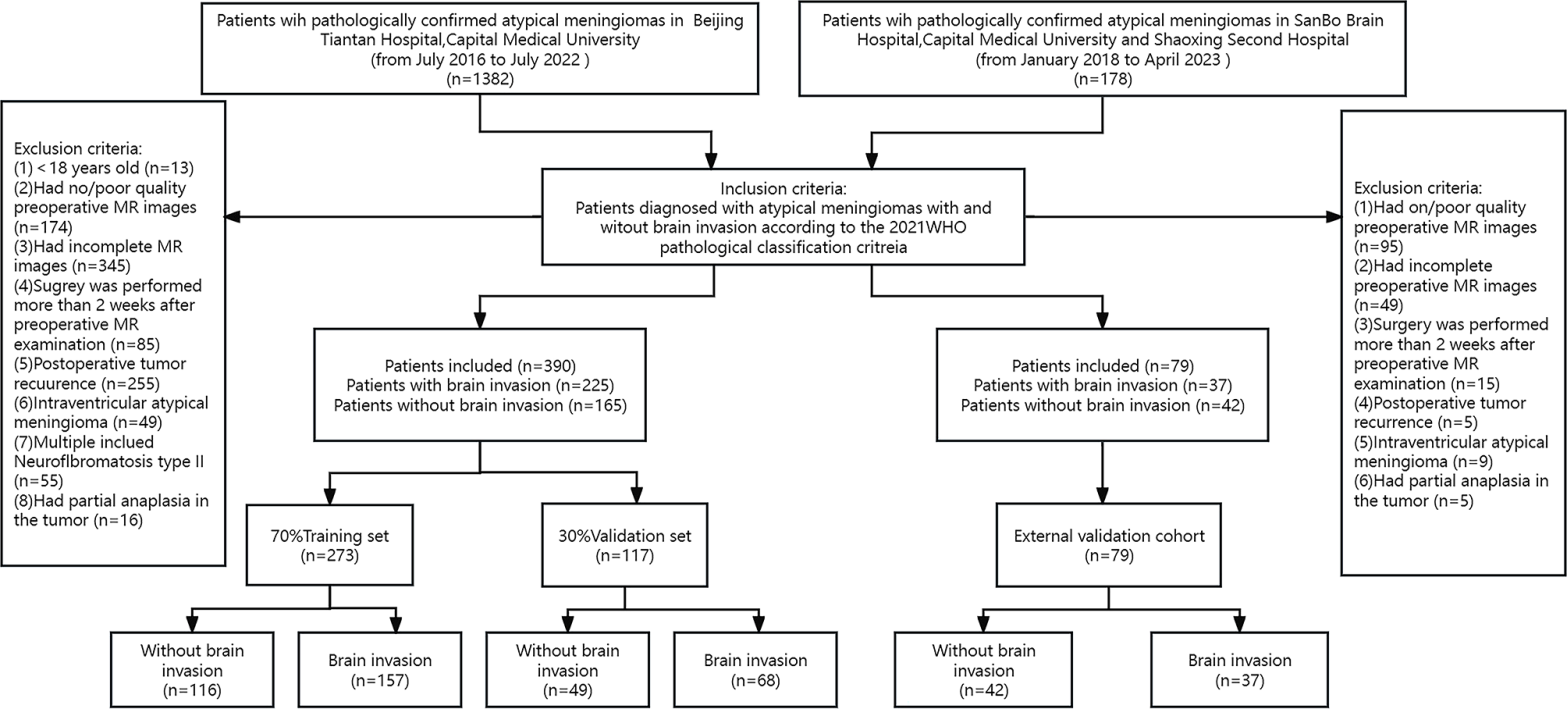
Workflow of patient selection WHO: World Health Organization

**Fig. 2:**
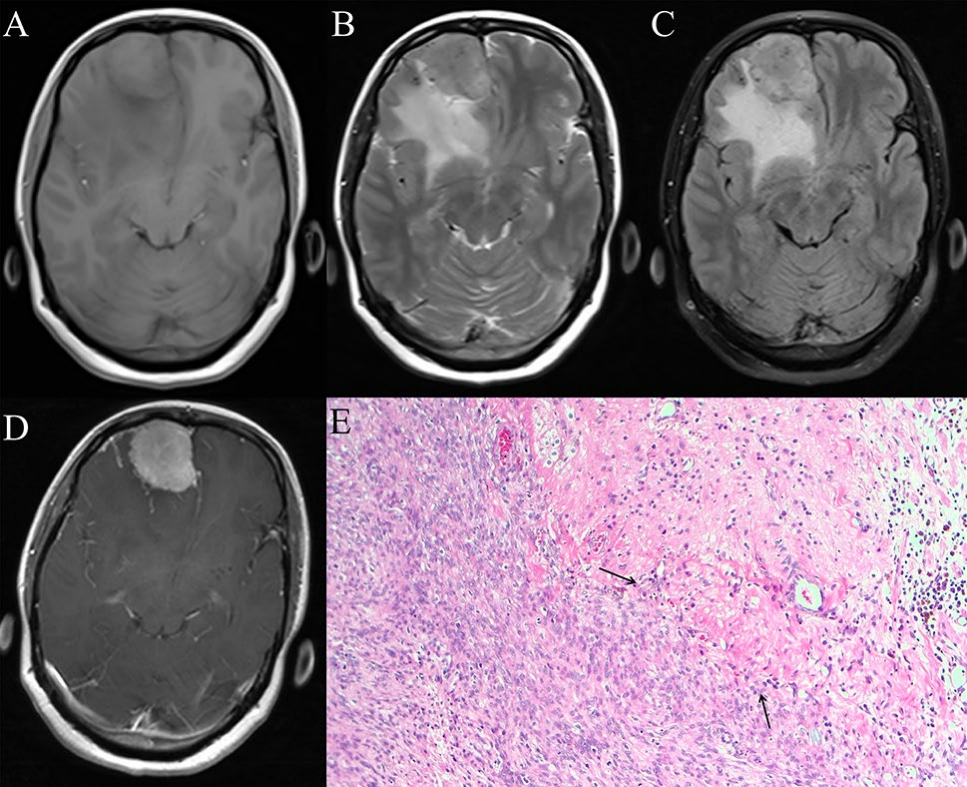
A 50-year-old female, presented with a tic of limbs, from centre 3. A mass is located in the right frontal lobe, which is diagnosed as atypical meningioma (AM) by pathological examination. (**A**) Axial T1-weighted MR images (T1C) show that the tumor and brain tissue are of equal intensity with unclear boundaries. (**B, C**) Axial T2-weighted MR images (T2) and T2-fluid attenuated inversion recovery images (FLAIR) show the equal intensity of the tumor and brain tissue, accompany by a large amount of peritumoral edema (PE), and the edema boundary is clearly displayed. (**D**) The T1C show that the tumor boundary is not smooth and the tumor-brain interface is blurred. (**E**) Pathological HE staining (10 × 10 magnification): the patient is diagnosed as AM with brain invasion (BI). Arrows show tumor tissue infiltrating into brain tissue

This retrospective study was approved by the Medical Ethics Committee of three medical centres, which waived the need for written informed consent from the patients. From the perspective of research design, we planed to include 3 radiomics scores (R-scores) and 6 clinical characteristics, to construct a logistic model. According to the 10 events per variable empirical principle, at least 90 positive and negative samples would be include into the training set.With a ratio of 7:3 for total sample, the total sample size should be greater than 258. From July 2016 to July 2022, 1382 patients with pathologically confirmed AM from medical centre 1 (Beijing Tiantan Hospital, Capital Medical University) were initially considered, and 390 patients with AM were finally included in the study. The detailed inclusion/exclusion criteria and enrolment process are shown in Fig. 1. In this study, the pathological samples were uniformly diagnosed with BI using light microscopy. BI was defined as the presence of adjacent intracerebral meningioma tissue without a separate connective tissue layer, with an appearance described as “an irregular tongue-like protrusion of tumor cells infiltrating the underlying brain parenchyma without an intervening pia mater” [2, 5], and the WHO classification was recorded; patients without BI were analysed by a combination of pathology reports and surgical records. Patients from centre 1 were randomly divided into a training cohort (*n* = 273) and an internal validation cohort (*n* = 117) at a ratio of 7:3 using the DeepWise Multimodal Research Platform version 2.5.1 (http://keyan.deepwise.com). A total of 79 AM patients from medical centre 2 (SanBo Brain Hospital, Capital Medical University) and centre 3 (Shaoxing Second Hospital) were collected as an external validation cohort. All data is based on one MRI which is the most recent MRI examination within 2 weeks before surgery. Representative images of multiparameter MRI and histopathology are shown in Fig. 2.

### MR image acquisition and segmentation

Two radiologists with more than 10 years of experience in neuroimaging independently interpreted the tumor location (skull base/non-skull base) on preoperative MRI. Inconsistent results were resolved by consultation; neither radiologist had any knowledge of the pathology results nor was involved in the subsequent analysis. A diagnostic radiologist with more than 10 years of experience used the open source software 3D-slice (version 5.0.3, https://www.slicer.org) to manually delineate the volume of interest (VOI) of the tumor along the border on contrast-enhanced T1-weighted MR (T1C) images and T2-weighted MR (T2) images independently, and both the tumor and PE were outlined on T2-fluid attenuated inversion recovery (FLAIR) images. After recording the volumes of tumors on the T1C and T2 images, to ensure the reliability of the data, the average of the two was taken as tumor volume (V_Tumor_), the volume outlined on the FLAIR images minus the volume of the tumor was considered as peritumoral edema volume (V_PE_), and V_PE_ was divided by V_Tumor_ to obtain the peritumoral edema index (PEI = V_PE_/V_Tumor_) [27, 28]. In our study, the clinical factors included clinical parameters (sex, age) and imaging parameters (tumor location, V_Tumor_, V_PE_ and PEI). The detailed scanning protocol and parameters are shown in Supplementary A.

### Image preprocessing

After manual separation of the tumor and PE using 3D Slicer (version 5.0.3, http://www.slicer.org), to reduce the variation in images acquired by different MR scanners, we normalised the T1C, T2 and FLAIR sequence images using z score normalisation after manually segmenting the VOI to obtain a standard normal distribution of image intensities while resampling all voxels to 1.0 × 1.0 × 1.0 mm^3^. Feature discretization was performed using bin width: 25. To ensure the accuracy of the VOI, 40 patients were randomly selected from the training cohort, and the VOI on the FLAIR sequence images were outlined again by the former and another radiologist in the same way as described above. Then, the inter-/intraclass correlation coefficients (ICCs) were used to evaluate the consistency between the VOI of the 40 patients and those outlined by the previous doctors. Features were extracted separately according to the outlined VOI to determine and assess the reliability between the VOI outlined by the same outliner at different times and between the VOI outlined by different outliners. High reproducibility was indicated when the mean ICCs of all features was > 0.75.

### Radiomics feature extraction and selection

We used the DeepWise Multimodal Research Platform version 2.5.1 (http://keyan.deepwise.com) with the Python PyRadiomics (version 3.0.1) and scikit-learn (version 0.22) packages as radiomics feature extraction and data analysis tools for this study. More details about feature extraction and selection are shown in Supplementary B.

### Model construction and validation

Using the radiomics score (R-score) of the tumor and PE features obtained from multiparameter MRI after least absolute shrinkage and selection operator (LASSO) regression screening and calculation, T1C, T2, FLAIR single models were developed in the training cohort. In the process of parameter adjustment, the optimal hyper-parameters were selected by using fivefold cross-validation and and grid search for the training cohort. Then, the R-scores of the T1C, T2 and FLAIR features were analysed by univariate and multivariate logistic regression, meaningful R-scores were selected and used to develop the fused radiomics model. The above four radiomics models were then independently verified in the internal and external validation cohorts. The subject ROC curves of each model were plotted, and the area under the ROC curve (AUC), accuracy (ACC), sensitivity (SEN), specificity (SPEC), positive predictive value (PPV) and negative predictive value (NPV) were calculated. The predictive performance of each model was evaluated and compared to determine the optimal model for predicting BI in AM patients.

The clinical characteristics of all included patients were analysed by univariate analysis and multivariate logistic regression to identify clinical risk factors significantly associated with a prediction of BI and construct clinical prediction models in the training cohort. Furthermore, the identified clinical factors were introduced into multivariate logistic regression along with the multiparameter R-scores to construct a comprehensive prediction model. The clinical, fused radiomics and comprehensive models were then compared in terms of their predictive efficacy for BI in AM, and the models were independently validated in the internal and external validation cohorts.

**Fig. 3:**
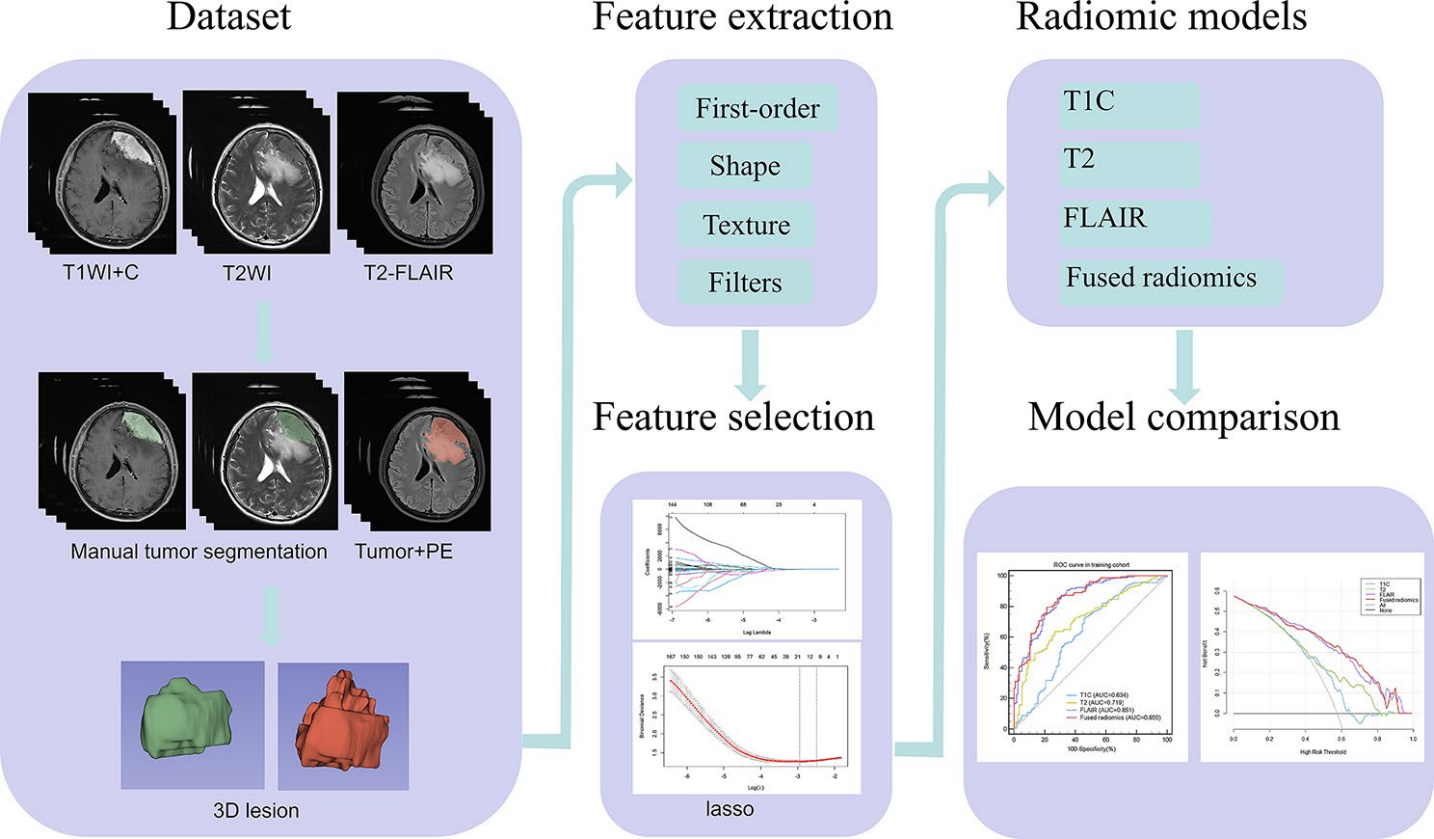
Flowchart of the radiomics study. First, data were collected, including contrast-enhanced T1-weighted MR images (T1C), T2-weighted MR images (T2) and T2-fluid attenuated inversion recovery (FLAIR) images, followed by manual tumor segmentation on the T1C and T2 sequences and manual tumor + PE segmentation on the FLAIR sequence. Second, the tumor and PE radiomics features were extracted, including first-order, shape, texture and filter features. Third, the least absolute shrinkage and selection operator (LASSO) was used to select the optimal features. Finally, T1C, T2, FLAIR and fused radiomics models were established to predict brain invasion in atypical meningioma patients, and their predictive performance was compared

Based on the results of the study, a clinical and radiomics nomogram of the optimal prediction model that was clinically meaningful was constructed, accurately predicting the likelihood of BI occurring in AM patients preoperatively. Figure 3 represents a radiomics flow chart of the study.

### Statistical methods

Continuous variables are expressed as the mean ± standard deviation, and categorical variables are expressed as frequencies (percentages). ANOVA (continuous variables) and the chi-square test (categorical variables) were used for data processing. The two-independent sample t test was used for variables with normal distributions, while the Wilcoxon signed-rank test was used for variables with a skewed distribution. The DeLong test was used to compare the ROC curves among the different models. *p* < 0.05 was considered to indicate statistical significance. The Deepwise Multimodal Research Platform version 2.5.1 (https://keyan.deepwise.com) was used as statistical analysis tool for this study.

## Results

### Clinical data

A total of 390 AM patients from centre 1 were enrolled in this study, including 225 (57.69%) patients with BI and 165 (42.31%) patients without BI. These patients were then divided into two cohorts at a ratio of 7:3, with 273 comprising the training cohort, including 157 patients with BI and 116 patients without BI, and 117 comprising the internal validation cohort, which included 68 patients with BI and 49 patients without BI. A total of 79 AM patients from centres 2 and 3 were used as the external validation cohort, including 37 patients with BI and 42 patients without BI. In the training cohort, patients with BI were statistically older (*p* = 0.046), but no such difference was observed in the internal and external validation cohorts. Sex and tumor location were not significantly different in the training and validation cohorts, and V_Tumor_ was statistically significant in the external validation cohorts and not significantly different in the training and internal validation cohorts. In the training, internal and external validation cohorts, V_PE_ (*p* < 0.01) and PEI (*p* < 0.01) were significantly higher in the patients with BI than in those without BI. The baseline characteristics of the patients in the training and validation cohorts are shown in Table 1.

**Table 1:** Baseline characteristics of atypical meningioma patients in the training and validation cohorts

Variable	Training cohort (*N*=273)		*P* value	Internal validation cohort (*N*=117)		*P* value	External validation cohort (*N*=79)		*P* value
	Noninvasion (*N*=116)	Invasion (*N*=157)		Noninvasion (*N*=49)	Invasion (*N*=68)		Noninvasion (*N*=42)	Invasion (*N*=37)	
Sex, (No., %)			0.458			0.408			0.105
Female	62 (53.4%)	91 (58.0%)		29 (59.2%)	35 (51.5%)		28 (66.7%)	18 (48.6%)	
Male	54 (46.6%)	66 (42.0%)		20 (40.8%)	33 (48.5%)		14 (33.3%)	19 (51.4%)	
Age (years, mean±SD)	51.78±13.87	55.09±13.52	0.046^*^	53.92±12.53	53.77±12.87	0.949	54.79±13.95	57.30±13.762	0.424
Location (No., %)			0.494			0.645			0.263
Non-skull base	72 (62.1%)	93 (59.2%)		26 (53.1%)	39 (57.4%)		22 (52.4%)	24 (64.8%)	
Skull base	44 (37.9%)	64 (40.8%)		23 (46.9%)	29 (42.6%)		20 (47.6%)	13 (35.1%)	
V_Tumor_ (cm^3^, P_25_,P_75_)	31.22 (17.04-61.88)	36.16 (19.50-71.64)	0.903	33.55 (16.08-59.37)	35.07 (18.54-72.02)	0.454	27.96 (9.29-46.66)	60.93 (28.50-82.37)	0.002^**^
V_PE_ (cm^3^, P_25_,P_75_)	8.828 (0.37-34.49)	65.66 (25.68-102.20)	<0.001^**^	7.01 (0.79-33.90)	47.97 (23.19-115.01)	<0.001^**^	3.86 (0.09-36.55)	47.84 (19.40-88.06)	<0.001^**^
PEI (P_25_,P_75_)	0.24 (0.02-0.78)	1.57 (0.72-2.88)	<0.001^**^	0.17 (0.02-0.64)	1.42 (0.47-3.66)	<0.001^**^	0.15(0.01-0.86)	0.84 (0.42-1.39)	0.002^**^

* *p*<0.05, ** *p*<0.01 V_Tumor_, Tumor volume; V_PE_, Peritumoral edema volume; PEI, Peritumoral edema index

### Clinical predictors of BI

Sex, V_PE_ and PEI were included in the multivariate logistic regression analysis (Supplementary C), showing that V_PE_ (*p* < 0.001, OR = 1.018, 95%CI: 1.008–1.027) and PEI (*p* = 0.035, OR = 1.333, 95%CI: 1.020–1.743) were significantly different between patients with and without BI and positively correlated with BI. After including V_PE_ and PEI, the clinical model achieved AUCs of 0.818 (95%CI: 0.765–0.870), 0.796 (95%CI: 0.703–0.857) and 0.773 (95%CI: 0.666–0.881) were obtained in the training, internal, and external validation cohorts, respectively.

### Selection of radiomics features

A total of 1409 T1C tumor features, 1409 T2 tumor features, and 1409 FLAIR tumor and PE features were extracted in this study. The ICCs of the outlines generated using the FLAIR images were excellent, with ICCs of 0.894 ± 0.175 and 0.850 ± 0.182, respectively. After Pearson correlation analysis and LASSO regression analysis, nine features from FLAIR, three features from T2 and two features from T1C images were selected. The heatmap of the Pearson correlation analysis for the selected features is shown in Supplementary D. As shown in the figure, the correlation between the selected radiomics features was low, and the feature redundancy was minimal. The analysis and weights of the selected radiomics features are shown in Table 2, which also shows that tumor and PE radiomics features based on the FLAIR sequence were predominant.

**Table 2:** Statistics of all selected radiomics features

Feature	Coef	Mean	Standard deviation	*P* value	Test
original_shape_Sphericity_Flair	0.1527	0.5588	0.1089	<0.001**	t
log-sigma-1-0-mm-3D_glszm_GrayLevelNonUniformity_Flair	0.6828	57.5973	41.7664	<0.001**	W
log-sigma-3-0-mm-3D_glszm_ZoneEntropy_Flair	0.2371	3.9044	0.6087	<0.001**	W
log-sigma-2-0-mm-3D_glszm_ZoneEntropy_Flair	0.5451	3.9873	0.5339	<0.001**	W
log-sigma-1-0-mm-3D_glrlm_RunEntropy_Flair	-0.5881	3.9082	0.1374	<0.001**	W
wavelet-LLL_glszm_ZonePercentage_Flair	-0.5744	0.0001	0.0002	0.029*	t
log-sigma-1-0-mm-3D_glcm_Correlation_Flair	0.5867	0.4739	0.0404	<0.001**	W
wavelet-HLL_gldm_DependenceEntropy_Flair	0.4603	5.1823	0.0610	<0.001**	W
wavelet-LLH_glrlm_LongRunLowGrayLevelEmphasis_Flair	0.4322	10.3298	1.8539	<0.001**	t
log-sigma-5-0-mm-3D_gldm_LargeDependenceHighGrayLevelEmphasis_T2	0.2854	1102.4890	326.3752	<0.001**	W
log-sigma-5-0-mm-3D_firstorder_90Percentile_T2	0.2267	0.1624	0.1118	<0.001**	t
log-sigma-2-0-mm-3D_glcm_Imc2_T2	0.6242	0.7178	0.0309	<0.001**	W
log-sigma-1-0-mm-3D_glcm_MCC_T1C	0.2214	0.4755	0.0383	<0.001**	W
log-sigma-1-0-mm-3D_glcm_ClusterProminence_T1C	0.2278	0.7736	0.0376	<0.001**	t

W, Wilcoxon test; t, t test. * *p*<0.05, ** *p*<0.01

### Construction and comparison of radiomics models

**Fig. 4:**
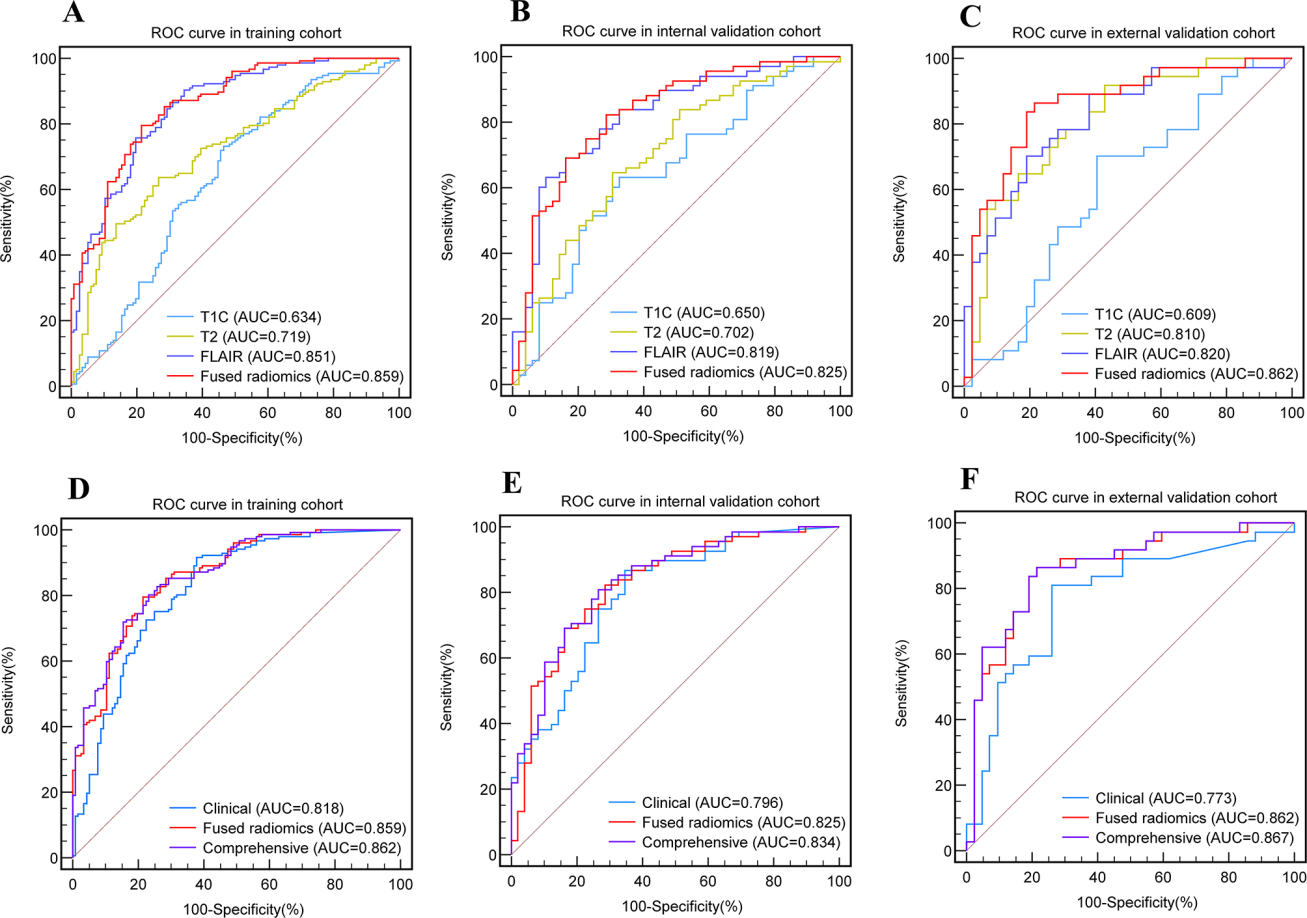
Comparison of the ROC curves of the different models (**A, B, C**) ROC curves of the different radiomics models in the training, internal validation and external validation cohorts. The fused radiomics model showed the best diagnostic efficacy among them, with an AUC of 0.859 in the training cohort (**A**), 0.825 in the internal validation cohort (**B**) and 0.862 in the external validation cohort (**C**). (**D, E, F**) ROC curves of the clinical, fused radiomics and comprehensive models in the training, internal and external validation cohorts. The comprehensive model showed the best diagnostic efficacy among these three models, with an AUC of 0.862 in the training cohort (**D**), 0.834 in the internal validation cohort (**E**) and 0.867 in the external validation cohort (**F**)

**Fig. 5:**
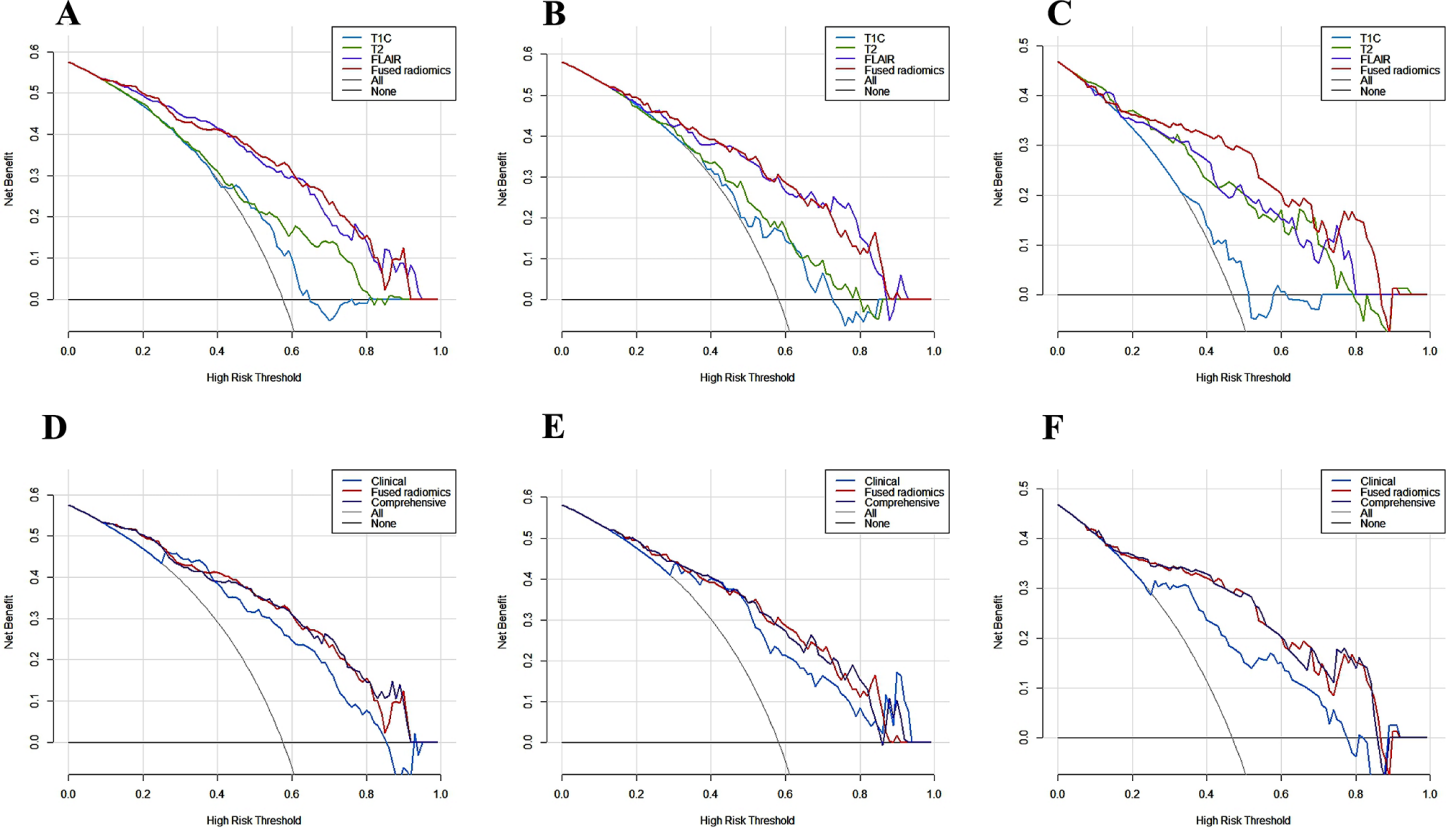
Decision curve analysis for the different models (**A, B, C**) Decision curves of the T1C, T2, FLAIR and fused radiomics models in the training, internal validation and external validation cohorts. (**D, E, F**) Decision curves of the clinical, fused radiomics and comprehensive models in the training, internal validation and external validation cohorts

The R-scores of the T1C, T2, and FLAIR (T1C-score, T2-score and FLAIR-score) tumor and PE radiomics features were calculated using the corresponding formulae, and multivariate logistic regression was used to construct the T1C, T2, FLAIR and fused radiomics models separately. A comparison of the models is shown in Fig. 4**(A-C)**. A comparison of the decision curves of the models is shown in Fig. 5**(A-C)**.

The results showed that the AUC of the model built from the tumor and PE radiomics features extracted from the FLAIR sequence in predicting BI in AM was significantly higher than that of the models based on the T2 and T1C tumor features in the training cohort (FLAIR model AUC = 0.851, 95%CI: 0.806–0.897), internal validation cohort (AUC = 0.819, 95%CI: 0.742–0.898) and external validation cohort (AUC = 0.820, 95%CI: 0.728–0.913). Furthermore, there was a significant difference (DeLong test, *p* < 0.05) among the AUCs of the three models; ranked according to *p* value, FLAIR > T2 > T1C.

The T1C-score, T2-score, and FLAIR-score were included in univariate and multivariate logistic regression analyses (Supplementary E). The results of multiple logistic regression showed that the T2-score and FLAIR-score were significantly different between patients with and without BI (*p* = 0.005 and *p* < 0.001, respectively) and positively correlated with BI. The T1C-score was not significantly different between the two groups (*p* = 0.609); therefore, only the T2-score and FLAIR-score were analysed by multivariate logistic regression to construct a fused radiomics model. The AUC of the fused radiomics model was higher than that of the FLAIR model alone in the training cohort (AUC = 0.859, 95%CI: 0.815–0.902), internal validation cohort (AUC = 0.825, 95%CI: 0.752–0.904), and external validation cohort (AUC = 0.862, 95%CI: 0.777–0.946). In addition, the ACC, SPEC, PPV and NPV of the fused radiomics model were higher than those of the single radiomics models in both the training and validation cohorts.

### Comparison of clinical, fused radiomics and comprehensive models

**Fig. 6:**
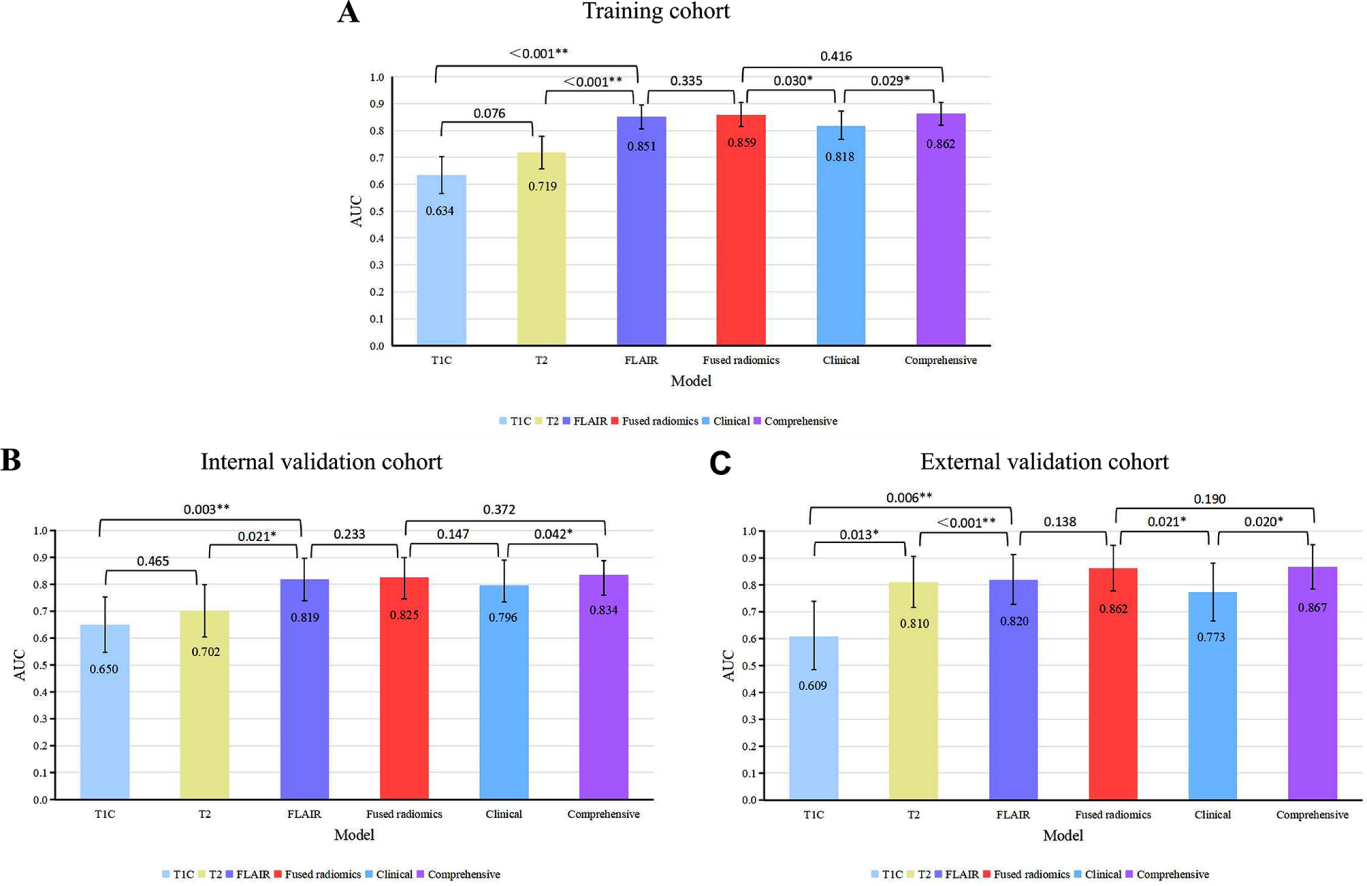
Comparison of AUC among the radiomics, clinical and comprehensive models in the training, internal and external validation cohorts. *p* < 0.05 was considered to indicate statistical significance. * *p* < 0.05, ** *p* < 0.01

Combining V_PE_, PEI, T2-score and FLAIR-score, a comprehensive model was constructed using multivariate logistic regression. The ROC curves of the six models are shown in Fig. 4, and the decision curves are shown in Fig. 5. The AUCs, ACCs, SENs, SPECs, PPVs and NPVs of the six models are shown in Table 3. The comparison of AUC among the clinical, radiomics and comprehensive models are shown in Fig. 6.

The results show that the comprehensive model was significantly superior to the clinical model (*p* = 0.030 in the training cohort, *p* = 0.021 in the external validation cohort) and had the highest AUC in predicting BI in AM patients, superior to that of the clinical model (*p* = 0.029 in the training cohort, *p* = 0.042 in the internal validation cohort and *p* = 0.020 in the external validation cohort) but not significantly different from that of the fused radiomics model (*p* > 0.05), which had the highest ACC, SEN, PPV, NPV and AUC in both the training and validation cohorts.

**Table 3:** Diagnostic performance of the radiomics signature, clinical and comprehensive models in the training and validation cohort

Model	Training cohort							Internal validation cohort								External validation cohort							
	ACC	SEN	SPEC	PPV	NPV	AUC	95%CI		ACC	SEN	SPEC	PPV	NPV	AUC	95%CI		ACC	SEN	SPEC	PPV	NPV	AUC	95%CI
**T1C**	0.604	0.605	0.603	0.674	0.530	0.634	0.565-0.703		0.641	0.618	0.674	0.724	0.559	0.650	0.548-0.752		0.544	0.526	0.786	0.526	0.550	0.609	0.479-0.732
**T2**	0.667	0.701	0.621	0.714	0.605	0.719	0.659-0.780		0.746	0.647	0.694	0.746	0.586	0.702	0.605-0.799		0.722	0.730	0.714	0.692	0.750	0.810	0.714-0.905
**FLAIR**	0.769	0.777	0.759	0.813	0.715	0.851	0.806-0.897		0.744	0.750	0.734	0.797	0.672	0.819	0.742-0.898		0.743	**0.865**	0.619	0.667	0.839	0.820	0.728-0.913
**Fused radiomics**	0.788	0.796	0.776	0.825	0.738	0.859	0.815-0.902		0.752	0.765	0.776	0.820	0.679	0.825	0.752-0.904		0.810	0.838	0.786	0.775	0.846	0.862	0.777-0.946
**Clinical**	0.722	0.661	**0.783**	0.814	0.639	0.818	0.765-0.870		0.650	0.544	**0.796**	0.787	0.575	0.796	0.703-0.857		0.532	0.622	0.738	0.676	0.689	0.773	0.666-0.881
**Comprehensive**	**0.795**	**0.815**	0.767	**0.826**	**0.754**	**0.862**	0.819-0.905		**0.761**	**0.779**	0.706	**0.833**	**0.735**	**0.834**	0.780-0.908		**0.823**	0.838	**0.810**	**0.795**	**0.850**	**0.867**	0.785-0.950

**ACC**, accuracy; **SEN**, sensitivity; **SPEC**, specificity; **PPV**, positive predictive value; **NPV**, negative predictive value; **AUC**, area under the curve; **T1C**, contrast-enhanced T1 weighted MR image; **T2**, T2 weighted MR image; **FLAIR**, T2-fluid attenuated inversion recovery

### Development of the nomogram

**Fig. 7:**
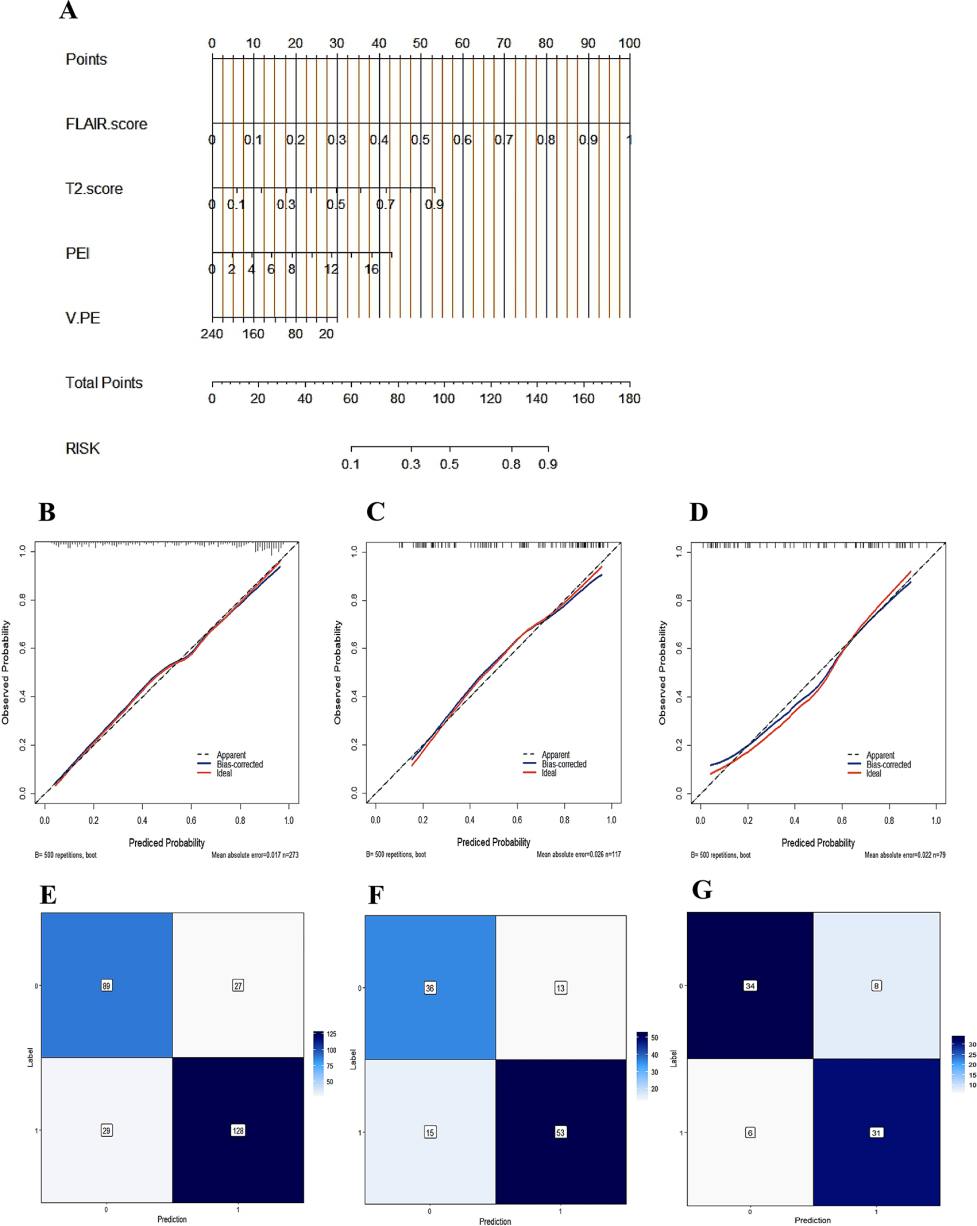
Nomogram of the comprehensive model and its calibration curves and confusion matrixes (**A**) The comprehensive model was constructed to develop the nomogram. (**B**, **C**, **D**) Calibration curves of the comprehensive model-based nomogram in the training, internal validation and external validation cohorts. (**E**, **F**, **G**) Confusion matrixes of the comprehensive model in the training, internal validation and external validation cohorts

A clinical and radiomics nomogram for preoperatively predicting the likelihood of BI in AM patients was constructed by combining V_PE_, PEI, T2-score and FLAIR-score, as seen in Fig. 7A, where each risk factor is labelled in quantitative form, and a total score is calculated based on the corresponding score for each risk factor for each AM patient to predict the risk of developing BI. The higher the total score is, the greater the risk of developing BI. Additionally, calibration curves were plotted for the training, internal and external validation cohorts to determine the predictive efficacy of the nomogram (Fig. 7B-D). The results show that the prediction curves are very close to the reference line, indicating strong predictive efficacy. Additionally, we show the confusion matrix of the comprehensive model (Fig. 7E-G), which reveals that the predicted false negatives and false positives are low in the training, internal validation and external validation cohorts.

## Discussion

In our study, the efficacy of MR radiomics features of AM tumor and PE in predicting the occurrence of BI in AM was compared between radiomics models built from different preoperative MRI sequences, a clinical model, a fused radiomics model and a comprehensive model constructed from the extracted MRI radiomics features and clinical features. The results showed that: (1) the AUC of the model built from the tumor and PE radiomics features extracted from FLAIR sequences in predicting BI in AM was significantly higher than that of the model based on the tumor features from the T2 and T1C sequences; (2) the radiomics features of PE play an important role in predicting BI; (3) PEI is an independent clinical risk factor in predicting BI in AM; and (4) the combined model (V_PE_, PEI, T2-score and FLAIR-score) showed the best performance in preoperatively predicting BI in AM patients in the training, internal validation and external validation cohorts.

Our study demonstrated that the incidence of BI in AM patients was 55.86%, which is higher than the 31.5% (95% CI 22.3–42.6%) before the revised criteria for AM were released [29–32] and in line with the findings from recent studies [8, 33]. The reason for this is that the new, revised criteria include BI as an independent, histological criterion for diagnosing AM. Formerly WHO grade 1 meningioma patients were included as having AM once the new BI-based criterion was applied to them, resulting in an increase of 1–10% in the incidence of AM [23, 34], as well as an increase in the proportion of BI in AM. In terms of clinical risk factors for BI in AM, our study found that patient sex, age, tumor location, and V_Tumor_ were not involved, consistent with the findings of several studies [8, 29]. In other studies, however, BI was more common in older, male meningioma patients and preferentially targeted areas other than the skull base [32, 35]. The possible reason for this inconsistency is that most of these other studies included all meningiomas and did not factor in the WHO classification. Our study showed that BI was associated with V_PE_ and PEI, with the probability of BI increasing with increased V_PE_ and PEI. Previous studies have suggested that PE is an independent predictor of BI [19, 20, 36], consistent with our study. Ong T et al. [20] studied BI in 60 meningioma patients and found that a larger PE may be associated with an increased incidence of BI. However, when controlling for tumor size, no statistically significant differences were found for PEI, in contrast to the results of our study.

Previous radiomics studies on predicting BI of meningiomas have primarily focused on analyzing the internal of the tumor and the tumor-brain interface [12, 26, 33, 36]. In this study, we developed a novel analysis by extracting the PE radiomics features and combining them with the internal features of the tumor. Nine features from FLAIR, three features from T2 and two features from T1C images were identified, demonstrating the clear preponderance of FLAIR sequence features. The results of the ROC analysis demonstrate that the AUC of the model constructed using tumor and PE radiomics features from the FLAIR sequence was significantly higher than that of the model based on the T2 and T1C tumor features in predicting BI in AM, which indicated the important role of both tumor and PE features in providing prediction-relevant information. One possible explanation for this finding is that when BI occurs in meningioma, changes also occur in the microenvironment surrounding the PE, and FLAIR sequences, which are sensitive to water in tissue, allow for clear delineation of edema boundaries. Another interesting result is that the FLAIR and T2 data proved to be more significant than the T1C data in predicting scores, this has also prompted us to pay more attention to the application of non-enhanced images in meningioma. In addition, the fused radiomics model combining FLAIR and T2 features outperformed both the single radiomics and clinical models. The nomogram constructed by combining V_PE_, PEI, T2-score and FLAIR-score demonstrated the best performance in predicting the occurrence of BI in AM patients preoperatively.

Li N et al. [33] conducted a study on 284 WHO grade 2 meningioma patients to predict BI based on tumor and tumor-brain border features.They found that combined clinical and conventional imaging indices had a slightly higher AUC than our study. However, the tumor-brain interface is not easy to delineate, and the inclusion of conventional imaging indicators was overrepresented, increasing the difficulty in implementing predictive models. Zhang J et al. [12] used T1C and T2 sequences to extract the radiomics features of meningioma. They developed a combined clinical-radiomics features nomogram for predicting BI in meningiomas, their study achieved an AUC of 0.857 in the training cohort and 0.819 in the validation cohort. However, it is worth noting that the majority of the study population in their research consisted of WHO grade 1 meningiomas, which may have introduced bias into the construction of the predictive models. Additionally, none of the results of the above studies had been externally validated. In our study, we included the largest number of AM cases compared to any published study, and we utilized a simple delineation for radiomics feature extraction and data from multiple centers. The AUC was 0.867 in the external validation cohort, indicating that our model is reliable and generalizable to other AM populations.

This study had several limitations. First, our study was retrospective, and there might be unavoidable selection bias. Second, the VOI was manually outlined using software, a time-consuming process that needs to be fully automated using better software to improve efficiency and avoid human error. Finally, as we retrospectively collected MR images from different centres, there were inevitable differences in equipment and scanning parameters. We standardised all the images to reduce the impact of these differences on the radiomics features, and good performance was obtained in the external validation cohort.

In conclusion, this study developed a predictive clinical and radiomics nomogram based on tumor and PE radiomics features extracted from multiparameter MRI, highlighting the important role that the radiomics features of PE play in predicting BI in AM patients. After further validation of data from multiple centers, the nomogram developed in this study could maximize the predictive accuracy for BI in AM patients in clinical practice and guide better clinical implementation of surgical protocols and personalized patient treatment, helping to improve the survival of AM patients.

### Electronic supplementary material

Below is the link to the electronic supplementary material.

Supplementary Material 1

## Data Availability

The datasets generated or analyzed during the study are available from the corresponding author on reasonable request.

## Declarations

### Data overlap

The datasets were constructed based on the collection of this study and had not been published repeatedly in previous studies.

## Abbreviations

ACC: Accuracy
AM: Atypical Meningioma
AUC: Area Under the Curve
BI: Brain Invasion
CNS: Central Nervous System
FLAIR: T2-Fluid Attenuated Inversion Recovery
ICCs: Inter-/Intraclass Correlation Coefficients
LASSO: Least Absolute Shrinkage and Selection Operator
NPV: Negative Predictive Value
PE: Peritumoral Edema
PEI: Peritumoral Edema Index
PPV: Positive Predictive Value
R-scores: Radiomics Scores
SEN: Sensitivity
SPEC: Specificity
T1C: Contrast-enhanced T1-weighted MR image
T2: T2-weighted MR image
VOI: Volume Of Interest
V_Tumor_: Tumor Volume
V_PE_: Peritumoral Edema Volume
WHO: World Health Organization
