# Supplementary material for: Multiparameter MRI-based radiomics nomogram for preoperative prediction of brain invasion in atypical meningioma:a multicentre study
Source: BMC Med Imaging. 2024 Jun 5;24:134. doi: 10.1186/s12880-024-01294-5 (PMC11154967; doi:10.1186/s12880-024-01294-5)
Supplement: Supplementary file 1 — Supplementary Material 1 [file 12880_2024_1294_MOESM1_ESM.docx]

**Supplementary A.**

After the pre-contrast scanning was finished, the dimeglumine gadopentetate was injected into the patient with a dose of 0.2 ml/kg via the antecubital venous. Once the injection was finished, the post-contrast scanning started.

The scanning parameters for the axial T2WI, FLAIR, and T1WI contrast enhancement of each MRI scanner.

The scanning parameters for the axial T2WI

| **MRI scanner** | **TR (ms)** | **TE (ms)** | **Slice thickness (mm)** | **FOV** |
| --- | --- | --- | --- | --- |
| GE Discovery MR750 | 6711 | 111 | 5.00 | 512×512 |
| GE Medical System Genesis Signa | 4291 | 127 | 4.00 | 512×512 |
| Siemens MAGNETOM Trio TimSystem | 5550 | 117 | 5.00 | 320×320 |
| Siemens MAGNETOM Verio  GE Singa HDe | 4500  4415 | 99  89 | 5.00  5.50 | 640×640  512×512 |
| Philips Ingenia | 2800 | 135 | 5.00 | 512×512 |
| Siemens Avanto | 5000 | 191 | 5.00 | 512×512 |

The scanning parameters for the axial FLAIR(T2-FLAIR)

| **MRI scanner** | **TR (ms)** | **TE (ms)** | **Slice thickness (mm)** | **FOV** |
| --- | --- | --- | --- | --- |
| GE Discovery MR750 | 8000 | 148 | 5.50 | 512×512 |
| GE Medical System Genesis Signa | 9000 | 121 | 4.00 | 512×512 |
| Siemens MAGNETOM Trio TimSystem | 6800 | 81 | 5.00 | 320×320 |
| Siemens MAGNETOM Verio  GE Singa HDe | 7500  9054 | 85  138 | 5.00  5.50 | 512×464  512×512 |
| Philips Ingenia | 4800 | 255 | 5.00 | 512×512 |
| Siemens Avanto | 8000 | 109 | 5.00 | 512×512 |

| **MRI scanner** | **TR (ms)** | **TE (ms)** | **Slice thickness (mm)** | **FOV** |
| --- | --- | --- | --- | --- |
| GE Discovery MR750 | 2804 | 19 | 5.00 | 512×512 |
| GE Medical System Genesis Signa | 2031 | 19 | 5.00 | 512×512 |
| Siemens MAGNETOM Trio TimSystem | 2000 | 9.8 | 5.00 | 512×432 |
| Siemens MAGNETOM Verio  GE Singa HDe | 1900  2378 | 9.4  10 | 5.00  5.00 | 512×496  512×512 |
| Philips Ingenia | 2000 | 20 | 5.00 | 512×512 |
| Siemens Avanto | 1773 | 17 | 5.00 | 512×512 |

The scanning parameters for the axial T1WI contrast enhancement

**Supplementary B.**

**Radiomics Feature extraction**

Based on the original and preprocessed images, we extracted tumor and PE radiomics features, including first-order features, texture features, shape-based features and higher-order features, including gray level cooccurrence matrix (GLCM), gray level size-zone matrix (GLSLM), gray level run-length matrix (GLRLM), gray level dependence matrix (GLDM), and neighbouring gray level-dependence matrix (NGTDM) features. We used five filters, respectively, the wavelet transform, laplacian of gaussian (LoG), two-dimensional local binary patterns (LBP2D), three-dimensional local binary patterns (LBP3D), Square. All other parameters remain in the default configuration. A total of 1409 radiomics features were extracted, and the data were normalised using the z score method as follows: z score = (x - mean)/SD. There was no missing data present.

**Feature screening**

First, we excluded features with unique values among the radiomics features extracted based on the tumor and PE. Second, the remaining features were separately subjected to Pearson correlation analysis and features with correlation coefficients above 0.9 were excluded. After the feature correlation analysis, the radiomics features based on tumor and PE were individually downscaled using the least absolute shrinkage and selection operator (LASSO) regression algorithm to select the most predictive features. The nonzero coefficients of the filtered features were calculated as the corresponding radiomics scores (R-scores).

Eq.A. R-score = 1 / (1 + exp(-logit))

T1C_logit=0.2214*log-sigma-1-0-mm-3D_glcm_MCC+0.2278*log-sigma-1-0-mm-3D_glcm_ClusterProminence + 0.0133

***T1C_R-score***=1/{1+exp{-(0.2214*log-sigma-1-0-mm-3D_glcm_MCC+0.2278*log-sigma-1-0-mm-3D_glcm_ClusterProminence + 0.0133)}}

T2_logit=0.2854*log-sigma-5-0-mm-3D_gldm_LargeDependenceHighGrayLevelEmphasis+0.2267*log-sigma-5-0-mm-3D_firstorder_90Percentile+0.6242*log-sigma-2-0-mm-3D_glcm_Imc2+ 0.0118

***T2_R-score***=1/{1+exp{-(0.2854*log-sigma-5-0-mm-3D_gldm_LargeDependenceHighGrayLevelEmphasis+0.2267*log-sigma-5-0-mm-3D_firstorder_90Percentile+0.6242*log-sigma-2-0-mm-3D_glcm_Imc2+0.0118)}}

FLAIR_logit=0.1527*original_shape_Sphericity+0.6828*log-sigma-1-0-mm-3D_glszm_GrayLevelNonUniformity+0.2371*log-sigma-3-0-mm-3D_glszm_ZoneEntropy+0.5451*log-sigma-2-0-mm-3D_glszm_ZoneEntropy+-0.5881*log-sigma-1-0-mm-3D_glrlm_RunEntropy+-0.5744*wavelet-LLL_glszm_ZonePercentage+0.5867*log-sigma-1-0-mm-3D_glcm_Correlation+0.4603*wavelet-HLL_gldm_DependenceEntropy+0.4322*wavelet-LLH_glrlm_LongRunLowGrayLevelEmphasis+ 0.0630

***FLAIR_R-score***=1/{1+exp{-(0.1527*original_shape_Sphericity+0.6828*log-sigma-1-0-mm-3D_glszm_GrayLevelNonUniformity+0.2371*log-sigma-3-0-mm-3D_glszm_ZoneEntropy+0.5451*log-sigma-2-0-mm-3D_glszm_ZoneEntropy+-0.5881*log-sigma-1-0-mm-3D_glrlm_RunEntropy+-0.5744*wavelet-LLL_glszm_ZonePercentage+0.5867*log-sigma-1-0-mm-3D_glcm_Correlation+0.4603*wavelet-HLL_gldm_DependenceEntropy+0.4322*wavelet-LLH_glrlm_LongRunLowGrayLevelEmphasis+ 0.0630)}}

**Supplementary C.**

Results of multiple logistic regression analysis

| Clinical variable | *P* value | OR | 95%CI |
| --- | --- | --- | --- |
| Age | 0.311 | 1.011 | 0.990-1.031 |
| V_PE_ | <0.001^**^ | 1.018 | 1.008-1.027 |
| PEI | 0.035^*^ | 1.333 | 1.020-1.743 |
| * *p*<0.05, ** *p*<0.01 | | | |

**Supplementary D**.  **Pearson correlation heatmaps of the selected radiomics features**


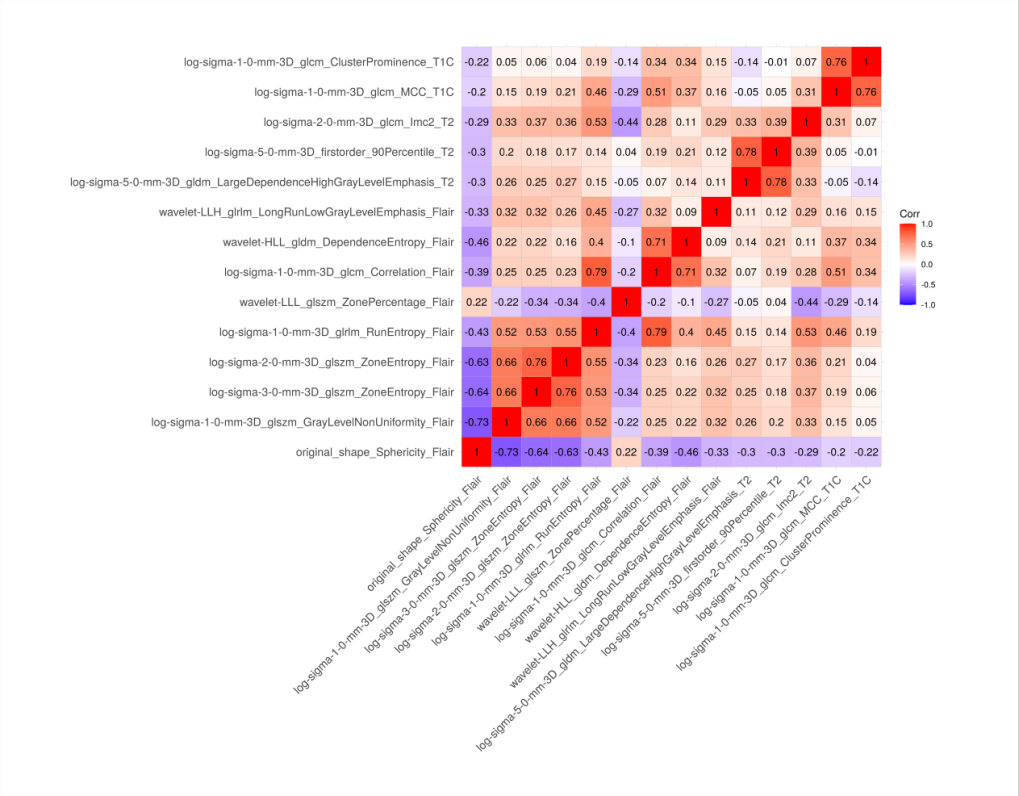


**Supplementary E.**

Results of univariate and multivariate logistic regression analyses

| R-score | Univariate |  | Multivariate |
| --- | --- | --- | --- |
|  | *P* value |  | *P* value |
| T1C | 0.001^**^ |  | 0.609 |
| T2 | <0.001^**^ |  | 0.005^**^ |
| FLAIR | <0.001^**^ |  | <0.001^**^ |
| * *p*<0.05, ** *p*<0.01 | | |  |
